# The Health-Related Quality of Life of Sarcoma Patients and Survivors in Germany—Cross-Sectional Results of a Nationwide Observational Study (PROSa)

**DOI:** 10.3390/cancers12123590

**Published:** 2020-11-30

**Authors:** Martin Eichler, Leopold Hentschel, Stephan Richter, Peter Hohenberger, Bernd Kasper, Dimosthenis Andreou, Daniel Pink, Jens Jakob, Susanne Singer, Robert Grützmann, Stephen Fung, Eva Wardelmann, Karin Arndt, Vitali Heidt, Christine Hofbauer, Marius Fried, Verena I. Gaidzik, Karl Verpoort, Marit Ahrens, Jürgen Weitz, Klaus-Dieter Schaser, Martin Bornhäuser, Jochen Schmitt, Markus K. Schuler, the PROSa Study Group

**Affiliations:** 1 Clinic and Polyclinic for Internal Medicine I, University Hospital Carl Gustav Carus, TU Dresden, 01307 Dresden, Germany; stephan.richter@uniklinikum-dresden.de (S.R.); martin.bornhaeuser@uniklinikum-dresden.de (M.B.); markus.schuler@helios-gesundheit.de (M.K.S.); 2 National Center for Tumor Diseases (NCT/UCC), 01307 Dresden, Germany; Leopold.Hentschel@uniklinikum-dresden.de (L.H.); christine.hofbauer@uniklinikum-dresden.de (C.H.); juergen.weitz@uniklinikum-dresden.de (J.W.); Klaus-Dieter.Schaser@uniklinikum-dresden.de (K.-D.S.); jochen.schmitt@uniklinikum-dresden.de (J.S.); 3 Division of Surgical Oncology & Thoracic Surgery, Mannheim University Medical Center, University of Heidelberg, 68167 Mannheim, Germany; peter.hohenberger@umm.de; 4 Interdisciplinary Tumor Center, Sarcoma Unit, University Medical Center Mannheim, 68167 Mannheim, Germany; bernd.kasper@umm.de; 5 Department of General Orthopedics and Tumor Orthopedics, University Hospital Munster, 48149 Munster, Germany; Dimosthenis.andreou@helios-gesundheit.de; 6 Sarcoma Center Berlin-Brandenburg, Helios Hospital Bad Saarow, 15526 Bad Saarow, Germany; daniel.pink@helios-kliniken.de; 7 Department of Internal Medicine C, University Hospital Greifswald, 17475 Greifswald, Germany; 8 Clinic for General, Visceral, and Pediatric Surgery, University Hospital Goettingen, 37075 Goettingen, Germany; jens.jakob@med.uni-goettingen.de; 9 Institute for Medical Biostatistics, Epidemiology and Informatic, University Hospital Mainz, 55131 Mainz, Germany; singers@uni-mainz.de; 10 Clinic for Surgery, University Hospital Erlangen, 91054 Erlangen, Germany; Robert.Gruetzmann@uk-erlangen.de; 11 Clinic for General, Visceral, and Pediatric Surgery, University Hospital Dusseldorf, 40225 Dusseldorf, Germany; Stephen.Fung@med.uni-duesseldorf.de; 12 Gerhard-Domagk-Institute for Pathology, University Hospital Munster, 48149 Munster, Germany; eva.wardelmann@ukmuenster.de; 13 German Sarcoma Foundation, 61200 Woelfersheim, Germany; Karin.Arndt@sarkome.de; 14 The Scientific Institute of Office-based Hematologists and Oncologists, 50676 Cologne, Germany; heidt@winho.de; 15 University Center for Orthopedics and Trauma Surgery, TU Dresden, 01307 Dresden, Germany; 16 Clinic and Polyclinic for Internal Medicine III/University-Centre for Tumor Diseases, University Hospital Mainz, 55131 Mainz, Germany; Marius.Fried@unimedizin-mainz.de; 17 Clinic for Internal Medicine III, University Hospital Ulm, 89081 Ulm, Germany; Verena.Gaidzik@uniklinik-ulm.de; 18 Inter-Local Joint Practice, Dres. Verpoort, Wierecky & Brandl, 20259 Hamburg, Germany; k.verpoort@t-online.de; 19 Medical Clinic II, University Hospital Frankfurt, 60590 Frankfurt am Main, Germany; marit.ahrens@kgu.de; 20 Department of Visceral, Thoracic and Vascular Surgery, University Hospital Carl Gustav Carus, TU Dresden, 01307 Dresden, Germany; 21 Center for Evidence-Based Healthcare, University Hospital Carl Gustav Carus, Technical University Dresden, 01307 Dresden, Germany; 22 Helios Hospital Emil von Behring, Department of Oncology, 14165 Berlin, Germany

**Keywords:** sarcoma, health-related quality of life, rare disease, observational study, clinically important restrictions and symptoms

## Abstract

**Simple Summary:**

Sarcomas are a rare cancer with many different subtypes. They can occur anywhere in the body and are treated in a multi-disciplinary manner. Large studies on the quality of life of sarcoma patients are rare, so little is known about how patients are doing compared to the general population and which groups of sarcoma patients are particularly affected by quality of life limitations. We assessed the quality of life of 1113 sarcoma patients from Germany. The majority were particularly restricted in their emotional functioning, physical functioning, and the exercise of everyday demands (role function). Many of them experienced pain (56%) and fatigue (51%). We found that patients with leg or bone sarcomas were especially affected by quality of life limitations. We also found that patients who received a retirement pension were less affected by quality of life restrictions than patients who had not retired.

**Abstract:**

Sarcomas are rare cancers with high heterogeneity in terms of type, location, and treatment. The health-related quality of life (HRQoL) of sarcoma patients has rarely been investigated and is the subject of this analysis. Adult sarcoma patients and survivors were assessed between September 2017 and February 2019 in 39 study centers in Germany using standardized, validated questionnaires (European Organization for Research and Treatment of Cancer Quality of Life Questionnaire (EORTC QLQ-C30)). Associated factors were analyzed exploratively using multivariable linear regressions. Among 1113 patients, clinically important limitations and symptoms were most pronounced in emotional (63%, 95% CI 60–66%), physical (60%, 95% CI 57–62%), role functioning (51%, 95% CI 48–54%), and pain (56%, 95% CI 53–59%) and fatigue (51%, 95% CI 48–54%). HRQoL differed between tumor locations with lower extremities performing the worst and sarcoma types with bone sarcoma types being most affected. Additionally, female gender, higher age, lower socioeconomic status, recurrent disease, not being in retirement, comorbidities, and being in treatment were associated with lower HRQoL. Sarcoma patients are severely restricted in their HRQoL, especially in functioning scales. The heterogeneity of sarcomas with regard to type and location is reflected in HRQoL outcomes. During treatment and follow-up, close attention has to be paid to the reintegration of the patients into daily life as well as to their physical abilities and emotional distress.

## 1. Introduction

Sarcomas are rare cancers, with about 7000 new cases per year in Germany [1] and an incidence of around 5 per 100,000 in Europe [2]. Five-year relative survival in 2000–2002 was 58% for soft tissue sarcomas and 62% for bone sarcomas [2]. Sarcomas form a heterogeneous group of tumors that includes a large variety of over 100 histological subtypes [3], can occur anywhere on the body, and whose therapy is based on complex and divergent treatment algorithms [4]. Preferred treatment modality for localized soft tissue sarcomas is surgery, often combined with (neo)adjuvant radiotherapy and/or (neo)adjuvant chemotherapy, depending on a variety of factors like tumor grade, histology and tumor location [4]. For bone sarcomas surgery is the first choice of treatment as well, in a variety of cases combined with chemotherapy and/ or radiotherapy [5]. If sarcomas are clinically not respectable radiotherapy or radiochemotherapy is used [4]. For gastrointestinal stromal tumors (GIST) surgery and a variety of tyrosine kinase inhibitors (TKI) are the preferred treatment options, depending on type of mutation, tumor size and other factors [6]. For locally advanced or marginally resectable extremity soft tissue sarcomas isolated limb perfusion (ILP) can be considered [7]. Regional hyperthermia is an option for localized high-risk soft tissue sarcomas in addition to neoadjuvant chemotherapy [8]. Un-resectable metastatic sarcomas are often treated with palliative chemotherapy and/or radiotherapy [4].

Sarcomas are often diagnosed late due to unspecific symptoms and rare occurrence [9]. Unplanned resections, result of misdiagnosing the tumor as a more common benign lesion, with a negative influence on the course of treatment are common [10,11]. Since 2018 in in Germany, it has been possible to have sarcoma centers certified as modules of an oncology center by the German Cancer Society [12]. Treatment at specialized centers is recommended by international guidelines [13]. In 2019, the “German Sarcoma Foundation” was founded, which is a joint organization of patients and physicians that is committed to improving the situation for sarcoma patients [14]. In 2017, the European Reference Network EURACAN (European Reference Network on Rare Adult Cancers (solid tumors)) for rare solid tumors in adults was established [15].

In addition to prolonged survival, cancer patients rate the improvement of quality of life as an important criterion for the treatment of tumor diseases [16,17]. However, the health-related quality of life (HRQoL) of sarcoma patients in the different stages of the disease is a rarely investigated topic worldwide [18]. This may be due to the rarity of the disease and the fact that sarcoma patients are treated at different facilities. A systematic review found a total of 20 publications between 2007 and 2017 [19]. The available publications refer to drug studies [20,21], specific localizations/ entities [22,23,24], single disease phases [25,26,27], or have small sample sizes [28]. The large study by van Eck et al. focused on the heterogeneity of surviving sarcoma patients in terms of tumor location [29].

This resulted in the following questions, which we addressed in an exploratory analysis:

(1) How is the HRQoL of sarcoma patients in Germany? How high is the percentage of patients with clinically important limitations and symptoms in the individual domains of HRQoL?
(2) Which factors are associated with selected HRQoL domains? Are there differences between sarcoma subtypes with respect to histology and location?

## 2. Results

### 2.1. Participation and Sample Description

**Figure 1:**
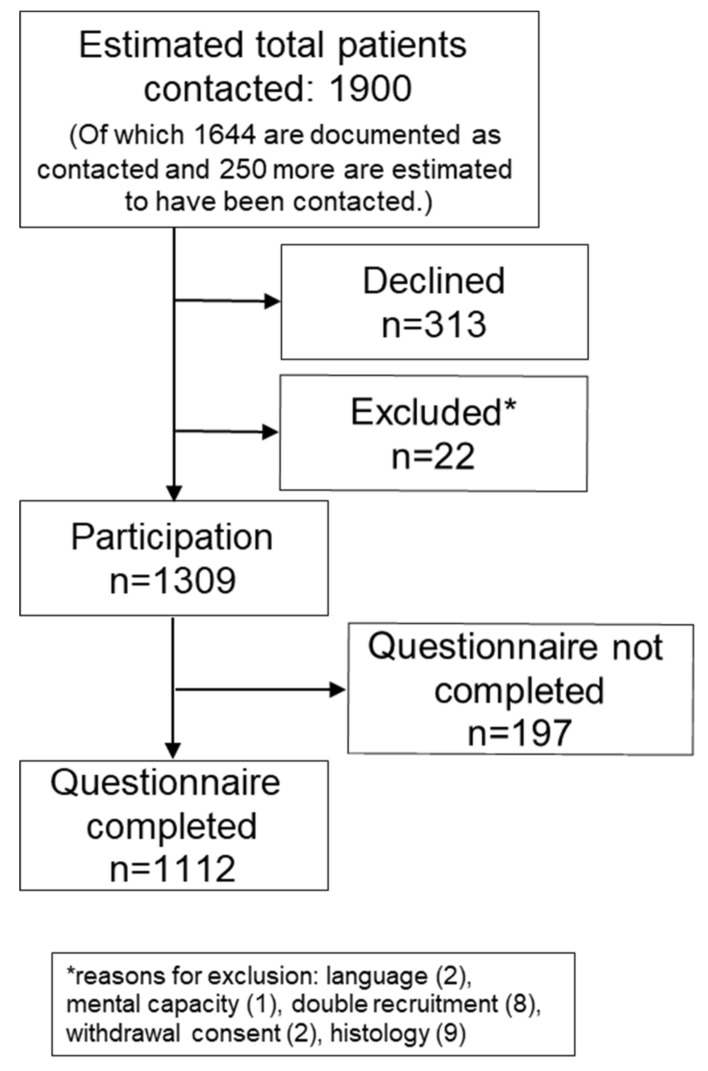
Study participation. Number of patients contacted overall had to be extrapolated from numbers of reporting study centers because not every study center documented contacted patients.

Approximately 1900 patients and survivors were approached and 1309 participated in the study (participation rate estimate: 69%). HRQoL data were available for 1113 patients and survivors (Figure 1). 70% of participants with HRQoL data had soft tissue sarcoma, 18% had bone sarcoma, and 12% had GIST. 33% of patients were under treatment (Table 1).

### 2.2. Health-Related Quality of Life

**Figure 2:**
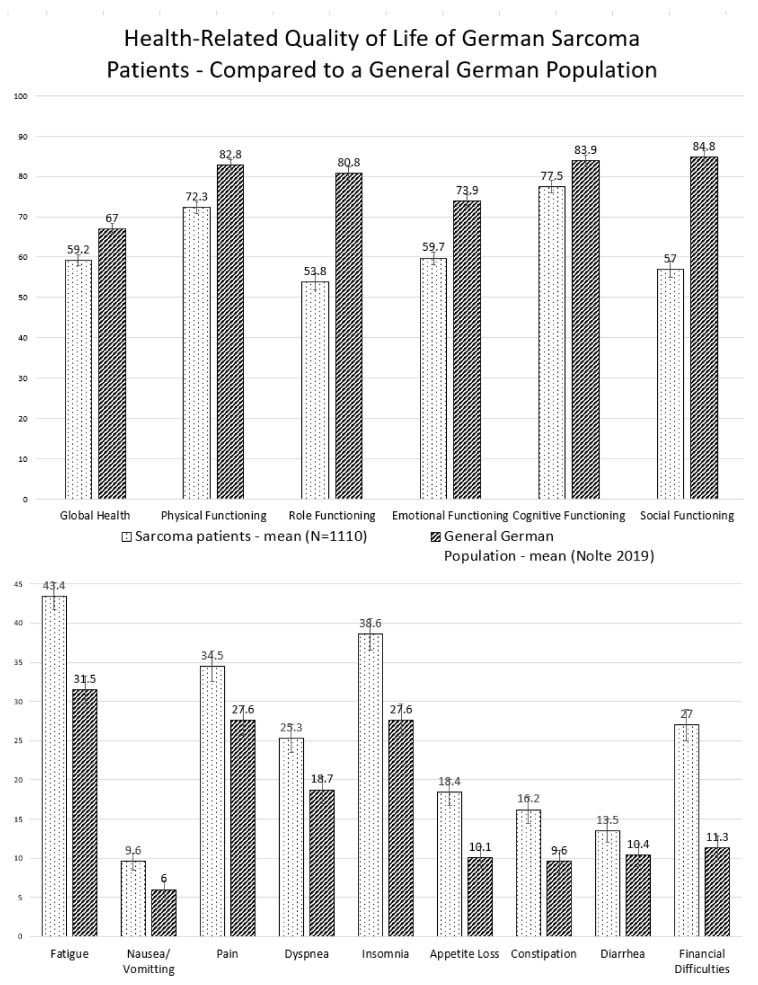
Age and gender standardized health-related quality of life of sarcoma patients and survivors. Mean values. Comparison with norm data from a German normal population (Nolte 2019). ↕95% confidence intervals. Large Differences: Role Functioning, Social Functioning; Medium/Moderate Differences: Financial Difficulties, Emotional Functioning; Small Differences: Fatigue, Dyspnea, Physical Functioning, Cognitive Functioning, Nausea/Vomiting, Pain, Insomnia, Appetite Loss, Diarrhea, Global Health, Constipation.

Mean global HRQoL was 59.5 out of a maximum of 100 points (Standard Deviation (SD) 22.7). Among the functioning scales, social (57.9, SD 33.1) and role functioning (54.3, SD 33.6) had the lowest values. Fatigue (43.2, SD 28.5), insomnia (38.5, SD 34.1), and pain (34.1, SD 31.6) showed the highest symptom loads. Gastrointestinal symptoms such as nausea and constipation were the least common. In an age- and gender-standardized comparison with a German normal population, all scales showed significant differences. Large differences were observed for role (27 points) and social functioning (27.8 points). Medium/ moderate differences in financial difficulties (15.7 points) and emotional functioning (14.2 points) were found (Figure 2).

### 2.3. Clinically Important Restrictions and Symptoms

Between 39% and 63% of patients had clinically important limitations in the functioning scales. The highest percentages were emotional (63%) and physical functioning (60%). The proportion of clinically important symptoms varied between 14% and 56%, with the lowest values for gastrointestinal symptoms such as constipation (14%) and lack of appetite (16%). The highest proportions were for pain (56%), fatigue (51%), and dyspnea (49%) (Table 2).

### 2.4. Stratified Analyses

Patients in palliative treatment showed significant differences compared to patients in curative situations in the majority of domains. Among the functional scales, the largest significant differences were found in social (57.9% vs. 42.3%), physical (70.8% vs. 55.6%), and role function (61.4% vs. 47.3%), and in symptom burden for dyspnea (63.7% vs. 44.6%), fatigue (65.1% vs. 46.4%), and diarrhea (40.0% vs. 21.8). When comparing sarcoma types, bone sarcoma patients were more restricted than soft tissue sarcoma patients, with the greatest differences were in role function (61.9% vs. 49.1%) and pain (65.0% vs. 54.0%). GIST patients were generally less affected, but reported diarrhea more often than soft-tissue sarcoma patients (44.6% vs. 23.9%) (Table 2).

### 2.5. Associated Factors in Multivariable Regression

#### 2.5.1. Socio-Demographics

Women showed significantly lower HRQoL values of trivial or small relevance than men in all eight domains. Higher age was significantly associated with worse values in five domains. Taking into account an age difference of 50 years, the differences in physical functioning were medium, in dyspnoea large. A higher Socioeconomic status (SES) was associated with better HRQoL in four domains. Comparing the lowest SES with the highest (3 vs. 21 points), the differences in pain are considered as large in physical functioning and as medium in general health. Patients in early retirement or collecting an old age pension had significantly better values in seven scales than those who were not retired The differences were medium in emotional functioning and small in the other domains (Table 3).

#### 2.5.2. Tumor Sites

In many of the evaluated tumor sites, we found a variety of significant and relevant differences. With lower limbs as the reference, patients with sarcomas of the upper limbs had better HRQoL outcomes of small and medium relevance in six scales. Patients with tumors of the head and neck scored better in five domains, in emotional functioning and pain those differences are considered as medium. Patients with abdominal or retroperitoneal sarcomas as well as thoracic sarcomas scored better in four domains. The latter performed worse in dyspnea. Patients with sarcomas of the pelvis reached a better outcome in role functioning. The differences were mainly found in physical, social, and role functioning and in pain symptoms (Table 3).

#### 2.5.3. Sarcoma Types

The comparison of the main nine sarcoma subtypes with liposarcoma as the most common as the reference showed that patients with one of the three main bone sarcoma types had the worst HRQoL outcomes. Patients with osteosarcoma and chondrosarcoma performed worse in six scales, those with Ewing sarcoma in five. The social functioning difference for chondrosarcoma patients is considered large. Differences between types of soft tissue sarcomas were observed as well. Patients with undifferentiated/ unclassified sarcomas scored worse in three scales, while those with synovial sarcomas had a poorer general health score. GIST patients had a better outcome in dyspnea. The differences were mainly found in general health, physical, social, and role functioning and in fatigue. No significant differences were found comparing patients with locally aggressive/rarely metastatic tumors with malignant ones (Table 3).

#### 2.5.4. Tumor-Related Factors

With low grade tumors as a reference, patients with high-grade tumors had lower HRQoL scores of small relevance in social functioning, while those with larger tumors showed no significant differences compared to those with smaller tumors. Metastases in the course of the disease had a negative effect on dyspnea. Patients who suffered from a recurrence of the tumor reported worse outcomes in seven domains. The differences here were trivial or small (Table 3).

#### 2.5.5. Disease and Treatment Status

The presence and number of comorbidities were associated with poorer HRQoL values in seven domains. Patients with stable disease or in partial remission had worse HRQoL outcomes in fatigue and social functioning, and those with progressive courses had a worse outcome in general health (reference: complete remission). Patients in treatment showed worse HRQoL outcomes in five domains compared to those not in treatment at the time of the survey. With “diagnosed in the last six months” as the reference, patients with a diagnosis more than 5 years ago reported better general health, social, and emotional functioning. Patients diagnosed between 1 and 2 years ago reported better outcomes in dyspnea and general health; those diagnosed between 2 and 5 years ago had better general health. No differences were found in physical functioning, pain, and fatigue (Table 3).

#### 2.5.6. Treatments Received

Treatments and combinations of treatments were significantly associated with HRQoL in two domains (reference: surgery alone). Patients who had received chemotherapy (CT) + surgery + radiotherapy (RT) showed poorer physical functioning and fatigue. Patients with surgery + CT experienced worse physical functioning. Patients with CT alone reported worse fatigue Table 3).

## 3. Discussion

### 3.1. Results in Context

Sarcoma patients and survivors are severely restricted in their health-related quality of life. Compared to the general population, role and social functioning are particularly strongly limited. The proportion of people with clinically important restrictions is consistently high, with a majority of patients reporting limitations in emotional, physical, and role functioning. The highest symptom burden is observed in pain, fatigue, and dyspnea. In comparison, the burden is rather low in gastrointestinal symptoms with the exception of GIST patients, who often receive tyrosine kinase inhibitors as long-term medication. The results of previous papers generally fit in well with the results of the PROSa study. Studies in more focused populations reported limitations in participation in daily life [22], physical limitations [23,24], and emotional stress [24,26,30]. Similar observations were also made regarding symptom burden, including fatigue [28,31], pain [25,30,31,32], shortness of breath [25,26,27], and insomnia [26,28].

The statistical analysis shows the association of socio-demographic factors as well as tumor and treatment-related factors with different HRQoL domains. Particularly noteworthy are the associations with socio-demographic factors that can be observed across almost all domains. While the association with age and gender is observed in almost all HRQoL studies [33], the positive correlation between HRQoL and early retirement/old age pension is not a general finding in oncology [34,35]. It seems possible that with the removal of occupational demands, mental and physical capacities are released, which ultimately has a positive effect on HRQoL. An indication of this is the strongly diminished role functioning in the study patients. Bone sarcomas occur particularly often in early adulthood, when the daily pressures in terms of work and child care are more prominent than in retirement.

Noteworthy as well and a strong indicator for the high heterogeneity of the disease are the differences we found in the most common sarcoma groups and tumor sites. All three bone sarcoma entities performed worse in the majority of analyzed domains than patients with liposarcomas, which we chose as a reference. Differences were observed within soft tissue sarcomas as well here patients with undifferentiated/unclassified sarcomas showed worse outcomes in some functioning scales. The soft tissue sarcoma groups are in part strongly diversified in itself—especially undifferentiated/unclassified as well as fibro/myofibroblastic sarcomas so that a more detailed analysis might show even more nuanced differences. The same is true for our analysis of different tumor sites. Patients with sarcomas of the upper extremities performed better than those with a tumor of the lower extremities. This might be in part an effect of the different functional restrictions related to the location of the tumor. However, differences were found in symptom scales as well especially in pain. Patients with tumors of the head and neck showed better outcomes in most functional scales and pain, and results for sarcomas of the trunk (thorax, abdomen) pointed in the same direction. The large study by von Eck et al in a population of Dutch sarcoma survivors found fewer differences with regard to tumor location than our analysis. The most affected group in that study (patients with sarcomas of the axial skeleton) cannot be directly depicted in our analysis as we chose a different classification system, but we see no indication that this group (if chosen) would have performed better in our analysis. Other differences between the study results—especially with regard to the results of patients with sarcomas of the lower extremities—are not easy to explain. It might be the case that the different study populations had an influence here [29].

Tumor-related factors (tumor size, grading) showed significant correlations in only a few or no HRQoL domains. This may be due to the fact that such information is not collected for all entities or in all situations. Another plausible hypothesis is that we included variables (tumor recurrence, disease status, treatment status) in the model that lie on the direct causal paths of the tumor-related factors und thus reduce the strength of the effect. We suspect the same mechanism with regard to treatment status, combined treatments, and metastasis by the time of study inclusion. The causal mechanisms and confounding structures are quite complex in this case (see supplement DAG). Treatment status and tumor recurrence are the two variables that showed significant and relevant associations in the most domains.

With regard to time since diagnosis, we found HRQoL improvements in only some domains, and these were mostly in the longer-term time spans. These results are to be interpreted with caution. The overall majority of our study participants were included during hospital/ practice visits. In survivors, clinical contacts become less frequent over time. It’s very likely that we have a sick survivor bias and included selected patients with more severe disease courses. That said, it is a worthwhile research question if our findings could be repeated in less selected populations.

### 3.2. Strengths and Limitations

The PROSa study is to our knowledge one of the largest studies on HRQoL in sarcoma patients and survivors worldwide. Patients from 39 hospitals and practices were included. The participating centers comprehensively represent the aspects of sarcoma treatment in Germany and have a large network of referring institutions. Previously published studies were often limited to subgroups specified by type, localization, or treatment, or were conducted in single centers and therefore did not allow inter group comparisons. Our analysis can provide an overview of the sarcoma patient population as it is presented at our study centers. The possible exception are sarcomas of the skin, which are often treated solely within the dermatology departments.

Since sarcomas present in an extremely heterogeneous clinical picture, the analysis of subtypes is even more necessary than usual. We were able to identify specific HRQoL issues in some of these groups, but we suspect that specific relations for example, concerning the influence of treatments or histological subtypes can only become visible in even more detailed analysis. It should also be noted that a symptom-specific questionnaire for sarcomere patients did not exist at the time of study execution. It can be assumed that in particular the limitations in the functioning scales are caused by a broad spectrum of site, tumor and treatment specific factors which present in variety of ways that could only be superficially captured by the generic questions of the European Organization for Research and Treatment of Cancer Quality of Life Questionnaire (EORTC QLQ-C30) [36,37].

The present study had a cross-sectional design. Causal conclusions are therefore not directly possible. It is also subject to selection bias. We see this possibility mainly on the level of the study centers. The majority of our patients were recruited in university hospitals and/or specialized centers and might so not representative for all sarcoma patients. Selection bias is also possible at the patient level. Here we suspect a sick survivor bias, as healthy survivors have less frequent contact with our recruiting study centers. The non-participant analysis, however, does not indicate any major systematic errors in that respect. The non-participant analysis is subject to the reservation that we have not been able to determine the exact number of non-participants and not every study center reported medical data on them. The possibility of undetected systematic confounding is inherent in observational studies, but we were able to measure a broad variety of potentially confounding variables.

## 4. Patients and Methods

To reach out to the broadest possible range of sarcoma treating facilities, data collection was preceded by extensive networking involving patient representatives, research communities, and professional societies. The prospective PROSa (Burden and medical care of sarcoma in Germany: Nationwide cohort study focusing on modifiable determinants of Patient-Reported Outcome measures in Sarcoma patients) cohort study (www.uniklinikum-dresden.de/prosastudie) was conducted nationwide between September 2017 and February 2019 in 39 study centers (NCT03521531; ClinicalTrials.gov). Of those 8 were office-based practices, 22 hospitals of maximum care and 9 other hospitals.

For the present analysis, cross-sectional data of adult patients and survivors with histologically proven sarcoma of any entity were analyzed (see Table S1). We excluded persons who were mentally or linguistically unable to complete questionnaires. Only participants with HRQoL data were analyzed.

Eligible patients and survivors were asked to participate at the participating study centers during visits (treatment, diagnose, aftercare) and sometimes by phone or letter. Participation required consent. The study was approved by the ethics committees of the Technical University of Dresden (EK1790422017) and the participating centers [38].

Data was collected by the study coordination center at University Hospital Dresden. HRQoL data and socio-demographic data were sent by the participants to the study coordination center by mail or online. Clinical information was submitted to the study coordination center online by the participating study centers using documentation forms. Data collection was performed using REDCap (Vanderbilt University, Nashville, United States) electronic data capture tools hosted at Technical University Dresden [39].

### 4.1. Variables

HRQoL was measured by means of the European Organization for Research and Treatment of Cancer Quality of Life Questionnaire (EORTC QLQ-C30) [40] This instrument measures global quality of life as well as 5 functioning and 9 symptom scales in values from 0 to 100 s. Higher values indicate a better quality of life for the functioning scales and a higher symptom burden for the symptom scales. Socioeconomic status (SES) was assessed using the Winkler Index [41]. The Winkler Index is a composite score which covers and quantifies three dimensions of SES: income, education and occupational prestige. On a scale of 3 to 21, a lower score means a lower SES.

In addition to age and gender, the clinical variables sarcoma type (undifferentiated/ unclassified, fibro/myofibroblastic, liposarcoma, leiomyosarcoma, osteosarcoma, chondrosarcoma, synovial sarcoma, Ewing sarcoma, GIST, other), tumor location (abdomen/retroperitoneum, thorax, pelvis, lower limbs, upper limbs, head & neck, back/spine, other sites (Table S2)), grading (G1, G2/G3), tumor size at diagnosis (T1, T2–T4, unknown + not applicable), tumor aggressiveness (locally aggressive + rarely metastatic, malignant), metastases up to the time of inclusion (yes, no, unknown), comorbidities at inclusion (0,1,2,3, > 3), disease status (complete remission, partial remission + stable disease, progression, unknown), treatment intention (palliative, curative), treatment status (yes, no), performed treatments (surgery alone, surgery + chemotherapy (CT), surgery + radiotherapy (RT), surgery + RT + CT, CT alone, other), time since diagnosis (<6 months, 6 < 12 months, 12 < 24 months, 24 < 60months, 60 months or more), and tumor recurrence (yes, no, unknown) were evaluated (for a tabular overview of the variables see Table 1).

### 4.2. Statistics

Continuous variables were evaluated by mean and standard deviation (SD) if normally distributed and by median and interquartile range (IQR) if not. Categorical variables were presented with absolute and relative frequencies. To contextualize results, an age and gender standardized comparison with reference values of the German normal population was performed [42]. The relevance of the differences was evaluated using reference values from Cocks and Osoba [43,44]. With these reference values, each scale difference can be classified as “small”, “moderate” and “large” (Osoba) or “trivial”, “small”, “medium” and “large” (Cocks).

We also reported stratified by sarcoma type and treatment intention the proportion of patients with clinically important symptoms and limitations (CIS + L) in the HRQoL domains using the thresholds of Giesinger et al. According to Giesinger et al. the concept of clinically important symptoms and limitations was developed to meet “the need for well-defined, valid thresholds for the absolute scores on the EORTC QLQ-C30 based on external criteria reflecting the clinical importance of a health problem. Clinical importance is defined as any aspect of a health problem that makes it relevant for the clinical encounter” [45].

We used a flow chart to report on study participation. A non-participant analysis was performed to estimate possible selection bias. The data of the non-participants, participants without HRQoL and the evaluated population were compared (Table S3).

Eight selected domains of EORTC QLQ-C30 (global quality of life, physical, social, emotional and role functioning, pain, fatigue, shortness of breath) were examined for associated factors. For this purpose, multivariable linear regressions were calculated and unstandardized regression coefficient (B), confidence intervals, p-values and R^2^ were evaluated for the whole model. Again, the relevance of the differences was evaluated using reference values from Cocks and Osoba [43,44].

Model variables were selected using direct acyclic graphs before analysis [46] (Figure S1).

To reduce the proportion of missing values in the SES, an imputation procedure was performed under the missing-completely-at-random assumption. The mean value of the overall index was calculated for the individual variable values of the three SES dimensions. If information on a single dimension was available in the participant’s data set, the overall mean value was imputed as the individual mean value of the participant.

Categorical variables were included in the analysis using dummy variables. To avoid multicollinearity, correlations and tolerance between the model variables were calculated before regression analyses. Correlations ≥ 0.7 and tolerance values ≤ 0.1 indicate strong multicollinearity problems. As a result, treatment intention was not included in the model and were evaluated in a stratified analysis.

Statistical analyses were performed with SPSS V.25 (IBM Corporation, Armonk, New York, NY, USA).

## 5. Conclusions

Compared to a German population, sarcoma patients and survivors are severely restricted in their health-related quality of life. The majority of them report clinically important restrictions in role, physical, and emotional functioning. Approximately half of the patients and survivors suffer from clinically important pain, fatigue, and dyspnea. Sociodemographic factors are associated with HRQoL limitations; the observed impact of reaching old age pension/early retirement for an increase in HRQoL is to be highlighted. We found a number of indications as to how the diversity of sarcoma disease manifests itself in HRQoL. Patients with sarcomas of the upper extremities, head and neck, of the abdominal/retroperitoneal region as well as the thoracic region performed better than those with sarcomas of the lower extremities. Patients with bone sarcomas had more severe HRQoL restrictions than soft tissue sarcoma patients, but there were also differences to be observed within the various soft tissue sarcoma entities.

During treatment and aftercare, increased attention should be paid to the frequent clinically important restrictions and symptoms of sarcoma patients especially with regard to role, physical, social and emotional functioning as well as to fatigue, pain and dyspnea.

## Figures and Tables

**Table 1:** Description of study population.

Variable	Value	N	%	Missing (Out of 1113)
Sex *	female	541	48.7	2
Age */** at study entry (mean: 52.6; SD: 16.3)	18–<40	185	16.6	1
	≥40–<55	264	23.7	
	≥55–<65	297	26.7	
	≥65–<75	233	21.0	
	≥75	133	12.0	
Time since diagnosis * (median: 2.3; IQR: 0.7–5.8)	0–<0.5 years	213	19.2	3
	≥0.5–<1 year	126	11.4	
	≥1–<2 years	165	14.9	
	≥2–<5 years	293	26.4	
	≥5 years	313	28.2	
Socio-economic status (Winkler-Index) */**/***	low (3.0–7.9)	113	10.6	44
	medium (8.0–13.8)	526	49.2	
	high (13.9–21)	430	40.2	
Early retirement/ old age pension *	yes	369	33.2	0
Sarcoma Types	soft tissue sarcoma	782	70.5	4
	bone sarcoma	197	17.8	
	GIST	130	11.7	
Sarcoma Types *	undifferentiated/unclassified	165	14.9	4 ****
	fibro-/myofibroblastic/fibrohistiocytic	130	11.7	
	liposarcoma	211	19	
	leiomyosarcoma	132	11.9	
	osteosarcoma	71	6.4	
	chondrosarcoma	64	5.8	
	synovial sarcoma	48	4.3	
	Ewing sarcoma	45	4.1	
	GIST	130	11.7	
	all other ****	113	11.2	
Tumor site *	abdomen/retroperitoneum	300	27.0	
	thorax	90	8.1	
	pelvis/urogenital	162	14.6	
	lower limbs	402	36.1	
	upper limbs	85	7.6	
	head & neck	35	3.1	
	back/spine	30	2.7	
	other/unknown	9	0.8	
Grading *	low grade (G1)	138	13.2	64 ****
	high grade (G2, G3)	603	57.5	
	not applicable *****	308	29.3	
T stage */****	T1	172	21.2	310 ****
	T2-T4	518	63.7	
	Tx****	13	1.6	
	Tumor without classification ****	110	13.5	
Aggressiveness of tumor *	locally aggressive + rarely metastatic	87	7.8	4
	malignant	1022	92.2	
Metastasis at study inclusion *	yes	369	37.8	138
Tumor recurrence *	no	801	71.4	20 ****
	yes	280	25.6	
	suspicion ****	12	1.1	
Treatment intention at study inclusion	palliative	261	24.0	26
Comorbidities */**	0	548	49.2	
	1	363	32.6	
	2	152	13.7	
	3	39	3.5	
	≥4	11	1.0	
Disease status *	complete remission	492	50.1	130
	partial remission + stable disease	330	33.5	
	progress	161	16.4	
Treatment status *	in treatment	369	33.4	7
Received treatment—surgery	yes	975	88.3	9
Received treatment—chemotherapy	yes	527	47.9	13
Received treatment—radiotherapy	yes	431	39.8	29
Received treatment—TKI	TKI − all	170	15.9	45
	TKI + surgery	144	11.4	
Combined treatments *	surgery alone	356	32.2	6 ****
	OP + CT	222	20.1	
	OP + RT	174	15.7	
	OP + CT + RT	222	20.1	
	CT alone	56	5.1	
	RT alone ****	9	0.8	
	CT + RT ****	26	2.3	
	no therapy (yet) ****	27	2.4	
	other therapies ****	15	1.4	

* variables in the model; ** continuous model variable; *** 35 of these were later imputed. **** aggregated in the model. ***** tumor not graded, no surgery, neoadjuvant therapy. SD: standard deviation; IQR: inter-quartile range; OP: Surgery; TKI: Tyrosine kinase inhibitors; RT: radiotherapy; CT: chemotherapy.

**Table 2:** Percentage of clinically important limitations and symptoms, stratified by treatment intention and sarcoma type.

Variable	All Patients (N = 1003–1100)		Curative (N = 818–825)		Palliative (N = 258–261)		Soft Tissue Sarcoma (N = 775–780)		Bone Sarcoma (N = 195–197)		GIST (N = 129–130)	
	%	95% CI	%	95% CI	%	95% CI	%	95% CI	%	95% CI	%	95% CI
Physical Functioning	59.5	56.5	55.6	52.1	**70.8**	64.8	58.8	55.2	67.0	60.2	50.8	41.9
		62.4		59.0		76.2		62.3		73.5		59.6
Role Functioning	50.7	47.7	47.3	43.8	**61.4**	55.2	49.1	45.5	**61.9**	54.8	42.6	34.0
						67.4		52.7		68.7		51.6
		53.7		50.7								
Emotional Functioning	62.7	59.8	61.2	57.8	67.7	61.6	63.2	59.7	61.4	54.2	61.5	52.6
		65.6		64.6		73.3		66.6		68.3		70.0
Cognitive Functioning	39.0	36.1	37.3	34.0	44.8	38.7	39.4	35.9	38.6	31.8	36.9	28.6
		42.0		40.7		51.1		42.9		45.8		45.8
Social Functioning	45.7	42.8	42.3	38.9	**57.9**	51.6	45.3	41.8	52.3	45.1	37.7	29.4
		48.7		45.7		63.9		48.9		59.4		46.7
Fatigue	50.9	48.0	46.4	43.0	**65.1**	59.0	51.2	47.6	53.3	46.1	45.0	36.2
						70.9		54.7		60.4		54.0
		53.9		49.9								
Nausea/Vomiting	27.5	24.9	23.4	20.6	**40.6**	34.6	25.4	22.4	29.9	23.7	35.4	27.2
												44.3
		30.2						28.6		36.9		
				26.5		46.8						
Pain	55.5	52.6	53.9	50.4	60.5	54.3	54.0	50.4	**65.0**	57.9	50.8	41.9
												59.6
		58.5				66.5		57.5		71.6		
				57.3								
Dyspnea	49.2	46.2	44.6	41.2	**63.7**	57.3	51.4	47.8	41.1	34.2	48.5	39.6
		52.2				69.6		55.0		48.3		57.4
				48.1								
Insomnia	35.3	32.4	35.4	32.1	36.8	30.9	34.7	31.4	33.5	27.0	41.1	32.5
		38.2		38.8		43.0		38.2		40.6		50.1
Appetite Loss	15.8	13.7	12.7	10.5	**24.5**	19.4	15.0	12.6	16.8	11.8	16.9	10.9
										22.7		24.5
		18.0				30.2						
				15.2				17.7				
Constipation	13.6	11.6	11.6	9.5	18.4	13.9	12.5	10.2	16.8	11.8	14.6	9.0
										22.7		21.9
		15.7		14.0		23.6		15.0				
Diarrhea	26.0	23.5	21.8	19.0	**40.0**	34.0	23.6	20.6	23.9	18.1	**44.6**	35.9
		28.7										53.6
						46.2		26.7		30.4		
				24.8								
Financial Difficulties	45.1	42.2	43.8	40.4	49.6	43.4	45.2	41.6	48.7	41.5	39.2	30.8
												48.2
		48.1				55.9				56.0		
				47.3				48.7				

Bold: Significant differences within groups (curative and soft tissue sarcoma as reference). Missing values not showed. 95% CI: Confidence interval for proportions.

**Table 3:** Results of the multivariable linear regression. # no difference; ## small difference; ### moderate difference (Osoba 1998). * trivial difference; ** small difference; *** medium difference; **** large difference (Cocks 2011); ^¶^ 35 values imputed. For continuous variables we compared: Age: 50-year difference. SES: 18-point difference; Comorbidities: 4-point difference. Significant Differences: bold. R^2^ = coefficient of determination; B= non-standardized regression coefficient (indicating a B point increase or decrease in the respective QoL scale); 95% CI (lower;upper): 95% confidence interval; *p* = *p*-value; na: not applicable; RT: radio therapy; CT: chemo therapy; SES: socioeconomic status.

Variable	Value	General Health (R^2^ = 0.16)			Physical Functioning (R^2^ = 0.19)			Social Functioning (R^2^ = 0.16)			Emotional Functioning (R^2^ = 0.08)		
		B	95% CI (l;u)	*p*	B	95% CI (l;u)	*p*	B	95% CI (l;u)	*p*	B	95% CI (l;u)	*p*
Sex	male vs. female	−2.91 *	−5.56; −0.27	**0.03**	−4.77 *	−7.53; −2.00	**<0.01**	−5.23 **	−9.07; −1.39	**0.01**	−6.62 ^##^	−9.83; −3.41	**<0.01**
Age	increase per year	−0.19 **	−0.33; −0.06	**0.01**	−0.31 ***	−0.45; −0.17	**<0.01**	−0.15	−0.34; 0.05	0.14	−0.08	−0.24; 0.08	0.34
SES ^¶^	increase per point	0.66 ***	0.31; 1.02	**<0.01**	0.92 ***	0.55; 1.29	**<0.01**	0.27	−0.24; 0.78	0.30	0.39	−0.04; 0.82	0.08
Old age pension/early retirement	no vs. yes	4.59 **	0.74; 8.43	**0.02**	5.45 **	1.42; 9.47	**0.01**	8.31 **	2.73; 13.88	**<0.01**	9.86 ^###^	5.18; 14.54	**<0.01**
Sarcoma Type	liposarcoma	reference											
	undifferentiated/unclassified	−4.24	−9.18; 0.71	0.09	−9.77 **	−14.95; −4.60	**<0.01**	−6.69	−13.85; 0.48	0.07	−6.56 ^##^	−12.56; −0.56	**0.03**
	fibro-/myofibroblastic	−4.76	−9.98; −0.46	0.07	−3.04	−8.51; 2.42	0.28	−0.75	−9.32; 6.83	0.85	−3.42	−9.75; 2.92	0.29
	GIST	0.46	−6.41; 7.34	0.90	−0.62	−7.83; 6.59	0.87	0.74	−9.24; 10.72	0.88	−0.07	−8.42; 8.28	0.98
	leiomyosarcoma	0.07	−5.17; 5.31	0.98	−1.20	−6.60; 4.27	0.67	2.21	−5.37; 9.78	0.57	−4.01	−10.34: 2.33	0.22
	osteosarcoma	−7.43 **	−14.50; −0.36	**0.04**	−15.01 ***	−22.46; −7.68	**<0.01**	−11.96 ***	−22.21; −1.71	**0.02**	−9.75 ^##^	−18.33; −1.18	**0.03**
	synovial sarcoma	−8.74 **	−16.09; −1.39	**0.02**	−7.67	−15.36; 0.23	0.051	−7.11	−17.78; 3.56	0.19	−3.40	−12.32; 5.52	0.46
	Ewing sarcoma	−13.24 ***	−21.50; −4.99	**<0.01**	−11.69 **	−20.33; −3.05	**0.01**	−12.85 ***	−24.83; −0.87	**0.04**	−6.39	−16.41; 3.63	0.21
	chondrosarcoma	−10.56 ***	−17.19; −3.94	**<0.01**	−15.82 ***	−22.76; −8.89	**<0.01**	−18.8 ****	−28.38; −9.15	**<0.01**	−6.10	−14.14; 1.95	0.14
	all other	−0.56	−6.23; 4.95	0.82	−5.66	−11.54; 0.22	0.06	−5.06	−13.19; 3.08	0.22	−4.37	−11.17; 2.43	0.21
Tumor Site	lower limbs	reference											
	abdomen/retroperitoneum	4.10	−0.28; 8.47	0.07	5.83 **	1.26; 10.40	**0.01**	7.17 **	0.83; 13.51	**0.03**	0.13	−5.18; 5.43	0.96
	thorax	3.76	−1.45; 8.97	0.16	8.38 **	2.90; 13.86	**<0.01**	8.94 **	1.37; 16.51	**0.02**	−2.38	−8.71; 3.96	0.46
	pelvis/urogenital	0.25	−4.00; 4.51	0.91	1.59	−2.86; 6.04	0.48	−0.43	−6.60; 5.74	0.89	0.31	−4.86; 5.48	0.91
	upper limbs	9.16 **	3.91; 14.39	**<0.01**	12.54 **	7.07; 18.02	**<0.01**	12.04 ***	4.74; 19.64	**<0.01**	1.99	−4.37; 8.35	0.54
	head & neck	7.29	−0.38; 14.96	0.06	10.78*	2.64; 18.81	**0.01**	16.30 ****	5.15; 27.44	**<0.01**	11.39 ^###^	2.07; 20.70	0.02
	back/spine	−1.17	−9.67; 7.33	0.79	3.71	−5.18; 12.60	0.41	0.27	−12.08; 12.61	0.97	1.57	−8.76; 11.89	0.77
	all other	15.34	−4.14; 34.81	0.12	6.86	−13.53; 27.25	0.51	5.60	−22.70; 33.89	0.70	3.04	−20.63; 26.71	0.80
Grading	G1	reference											
	G2/G3	−0.45	−4.87; 3.98	0.84	−3.44	−8.07; 1.18	0.15	−6.84 **	−13.27; −0.43	**0.04**	−1.58	−6.94; 3.79	0.57
	other (unknown/na)	0.56	−4.91; 6.03	0.84	−1.10	−6.84; 4.64	0.71	−5.45	−13.40; 2.50	0.18	−3.82	−10.47; 2.83	0.26
T-Stadium	T1	reference											
	T2-T4	1.06	−3.07; 5.18	0.62	−2.54	−6.84; 1.77	0.25	−4.86	−10.85; 1.13	0.11	−2.54	−7.55; 2.47	0.32
	other (unknown/na)	2.56	−1.95; 7.06	0.27	0.68	−4.02; 5.37	0.78	−2.93	−9.46; 3.60	0.40	−0.75	−6.21; 4.72	0.79
Aggressiveness Tumor	malignant vs. locally aggressive + rarely metastatic	2.96	−4.06; 9.99	0.41	−2.16	−9.51; 5.18	0.56	−4.34	−14.54; 5.86	0.40	−3.24	−11.78; 5.29	0.46
Metastasis till study inclusion	no	reference											
	yes	−1.86	−5.47; 1.75	0.31	−3.07	−6.85; 0.70	0.11	−3.83	−9.06; 1.41	0.15	−1.70	−6.08; 2.68	0.45
	unknown	−1.82	−6.18; 2.54	0.41	−2.14	−6.69; 2.41	0.36	0.86	−5.47; 7.20	0.79	−2.17	−7.47; 3.13	0.42
Comorbidities	increase per comorbidity	−2.78 ***	−4.43; −1.12	**<0.01**	−3.43**	−5.16; −1.69	**<0.01**	−3.41 ***	−5.81; −1.00	**0.01**	−1.90	−3.91; 0.07	0.07
Disease status	complete remission	reference											
	part. remission + stable disease	−3.17	−6.77; 0.43	0.08	−0.14	−3.91; 3.63	0.94	−5.51 **	−10.74; −0.28	**0.04**	−4.11	−8.48; 0.27	0.07
	progress	−7.98 **	−12.87; −3.09	**<0.01**	−2.24	−7.34; 2.86	0.39	−6.95	−14.03; 0.13	0.054	−5.32	−11.25; 0.61	0.08
	unknown	−0.39	−5.46; 4.69	0.88	0.68	−4.63; 5.99	0.80	−8.54 **	−15.91; −1.17	**0.02**	−4.25	−10.42; 1.91	0.18
Treatment status	no vs. yes	−6.32 **	−9.90; −2.75	**<0.01**	−5.03**	−8.76; −1.30	**0.01**	−5.90 **	−11.08; −0.71	**0.03**	−0.67	−5.01; 3.66	0.76
Combined Treatments	surgery alone	reference											
	surgery + CT	−0.39	−4.65; 3.88	0.86	−4.63**	−9.10; −0.16	**0.04**	−4.64	−10.84; 1.56	0.14	2.72	−2.46; 7.91	0.30
	surgery + RT	0.02	−4.32; 4.37	0.99	−2.35	−6.89; 2.20	0.31	−4.71	−11.02; 1.61	0.14	−0.40	−5.68; 4.88	0.88
	surgery + CT + RT	−1.17	−5.61; 3.27	0.61	−5.25**	−9.89; −0.61	**0.03**	−4.95	−11.39; 1.49	0.13	−2.72	−8.10; 2.67	0.32
	CT alone	−0.56	−6.57; 7.68	0.88	−6.94	−14.43; 0.55	0.07	−5.78	−16.13; 4.57	0.27	7.40	−1.32; 16.12	0.10
	all other	−1.81	−7.66; 4.04	0.54	−3.76	−9.86; 2.34	0.23	−4.31	−12.78; 4.16	0.32	−2.31	−9.40; 4.77	0.52
Time since diagnosis	0–<6months	reference											
	6–<12months	0.92	−4.26; 6.10	0.73	−2.89	−8.32; 2.54	0.30	−3.18	−10.70; 4.35	0.41	−0.08	−6.37; 6.22	0.98
	12–<24months	6.68 **	1.64; 11.72	**0.01**	−1.67	−6.93; 3.60	0.54	3.91	−3.41; 11.22	0.30	2.52	−3.62; 8.66	0.42
	24–<60months	8.67 **	3.89; 13.44	**<0.01**	0.91	−4.07; 5.90	0.72	6.25	−0.68; 13.18	0.08	2.69	−3.11; 8.49	0.36
	60 months or more	7.73 **	2.60; 12.87	**<0.01**	−1.88	−7.24; 3.49	0.49	9.54 **	2.09; 16.99	**0.01**	7.15 ^##^	0.91; 13.39	**0.03**
Tumor recurrence	no	reference											
	yes	−4.96 *	−8.43; −1.49	**0.01**	−3.67 *	−7.30; −0.04	**0.04**	−8.35 **	−13.38; −3.32	**<0.01**	−5.52 ^##^	−9.73; −1.31	**0.01**
	unknown	0.73	−7.75; 9.21	0.87	−2.80	−11.67; 6.09	0.54	2.27	−10.05; 14.59	0.72	−1.26	−11.75; 9.23	0.81
**Variable**	**Value**	**Role Functioning (R^2^ = 0.16)**			**Pain (R^2^ = 0.10)**			**Fatigue (R^2^ = 0.16)**			**Dyspnea (R^2^ = 0.14)**		
		**B**	**95% CI (l;u)**	**p**	**B**	**95% CI (l;u)**	**p**	**B**	**95% CI (l;u)**	**p**	**B**	**95% CI (l;u)**	**p**
Sex	male vs. female	−6.11 **	−10.01; −2.22	**<0.01**	4.16 *	0.36; 7.97	**0.03**	8.45 **	5.13; 11.76	**<0.01**	5.50 **	1.95; 9.05	**<0.01**
Age	increase per year	−0.20 **	−0.40; −0.004	**0.045**	0.12	−0.07; 0.31	0.22	0.26 ***	0.10; 0.43	**<0.01**	0.33 ****	0.15; 0.51	**<0.01**
SES^¶^	increase per point	0.45	−0.07; 0.97	0.09	−1.25 ****	−1.76; −0.74	**<0.01**	−0.59 **	−1.03; −0.15	**0.01**	−0.23	−0.71; 0.24	0.33
Old age pension/early retirement	no vs. yes	8.18 **	2.51; 13.85	**0.01**	−2.96	−8.49; 2.53	0.29	−8.82 **	−13.64; −4.00	**<0.01**	−6.78 **	−11.98; −1.62	**0.01**
Sarcoma Type	liposarcoma	reference											
	undifferentiated/unclassified	−9.74 **	−17.02; −2.47	**0.01**	5.14	−1.95; 12.38	0.16	4.54	−1.64; 10.73	0.15	1.13	−5.52; 7.77	0.74
	fibro-/myofibroblastic	−5.62	−13.31; 2.07	0.15	4.68	−2.83; 12.19	0.22	3.25	−3.29; 9.79	0.33	−0.53	−7.55; 6.49	0.88
	GIST	6.88	−3.26; 17.02	0.18	5.71	−4.18; 15.61	0.26	0.30	−8.33; 8.83	0.95	−9.76 ***	−18.98; −0.54	**0.04**
	leiomyosarcoma	1.95	−5.75; 9.63	0.62	−2.40	−9.92; 5.12	0.53	0.08	−6.46; 6.62	0.98	−2.56	−9.58; 4.46	0.48
	osteosarcoma	−13.79 **	−24.21; −3.38	**0.01**	8.94	−1.22; 19.11	0.09	10.25 **	1.40; 19.10	**0.02**	5.10	−4.37; 14.58	0.29
	synovial sarcoma	−8.72	−19.55; 2.12	0.12	4.69	−5.89; 15.27	0.38	5.05	−4.17; 14.26	0.28	7.45	−2.40; 17.23	0.14
	Ewing sarcoma	−13.50 **	−25.67; −1.33	**0.03**	8.67	−3.21; 20.55	0.15	11.94 **	1.60; 22.29	**0.02**	2.93	−8.13; 13.99	0.60
	chondrosarcoma	−19.55 ***	−29.31; −9.78	**<0.01**	14.78 ***	5.24; 24.31	**<0.01**	9.09 **	0.79; 17.34	**0.03**	1.43	−7.45; 10.32	0.75
	all other	−4.85	−13.11; 3.41	0.25	2.78	−5.29; 10.84	0.50	5.26	−1.76; 12.28	0.14	−0.40	−7.92; 7.11	0.92
Tumor Site	lower limbs	reference											
	abdomen/ retroperitoneum	9.12 **	2.68; 15.57	**0.01**	−8.81 **	−15.1; −2.52	**0.01**	−0.90	−6.28; 4.58	0.75	4.11	−1.76; 9.99	0.17
	thorax	9.53 **	1.87; 17.25	**0.02**	−12.21 **	−19.7; −4.71	**<0.01**	−1.33	−7.89; 5.21	0.69	8.33 ***	1.34; 15.31	**0.02**
	pelvis/urogenital	7.10 **	0.83; 13.37	**0.03**	−2.99	−9.11; 3.14	0.34	1.05	−4.28; 6.38	0.70	−2.12	−7.84; 3.59	0.47
	upper limbs	10.47 **	2.75; 18.19	**0.01**	−14.28 ***	−21.8; −6.74	**<0.01**	−7.41 **	−13.98; −0.85	**0.03**	−1.55	−8.56; 5.46	0.67
	head & neck	13.45 **	2.14; 24.77	**0.02**	−14.03 ***	−25.0; −2.98	**0.01**	−6.25	−15.89; 3.38	0.20	−4.45	−14.87; 5.98	0.40
	back/spine	−2.55	−15.09; 9.99	0.69	1.65	−10.59; 13.9	0.79	4.13	−6.53; 14.79	0.45	6.17	−5.21; 17.56	0.29
	all other	9.13	−19.62; 37.87	0.53	−25.55	−53.60; 2.51	0.07	0.27	−24.17; 24.71	0.98	−7.45	−33.54; 18.63	0.58
Grading	G1	reference											
	G2/G3	−4.71	−11.23; 1.81	0.16	2.82	−3.55; 9.19	0.39	3.83	−1.72; 9.37	0.18	0.26	−5.70; 6.21	0.93
	other (unknown/na)	−5.44	−13.52; 2.54	0.19	1.88	−6.00; 9.76	0.64	3.36	−3.51; 10.22	0.34	3.41	−3.95; 10.77	0.36
T-Stadium	T1	reference											
	T2–T4	−1.91	−7.98; 4.16	0.54	0.28	−5.65; 6.20	0.93	−2.17	−7.34; 3.00	0.41	−2.47	−8.02; 3.08	0.38
	other (unknown/na)	2.61	−4.01; 9.23	0.44	1.00	−5.37; 7.57	0.74	−6.27 **	−11.91; −0.63	**0.03**	−6.72 **	−12.79; −0.66	**0.03**
Aggressiveness Tumor	malignant vs. locally aggressive + rarely metastatic	1.14	−9.21; 11.50	0.83	8.44	−1.67; 18.54	0.10	−2.80	−11.61; 6.01	0.53	−3.71	−13.17; 5.74	0.44
Metastasis till study inclusion	no	reference											
	yes	−5.07	−10.38; 0.25	0.06	−0.11	−5.30; 5.08	0.97	4.49	−0.03; 9.02	0.052	7.81 **	2.96; 12.67	**<0.01**
	unknown	−2.19	−8.60; 4.22	0.50	1.64	−4.62; 7.89	0.61	2.92	−2.55; 8.39	0.30	2.31	−3.55; 8.17	0.44
Comorbidities	increase per comorbidity	−3.98 **	−6.42; −1.54	**<0.01**	2.69 **	0.30; 5.07	**0.03**	4.11 ***	2.03; 6.19	**<0.01**	4.44 ****	2.23; 6.66	**<0.01**
Disease status	complete remission	reference											
	part. remission + stable disease	−3.95	−9.26; 1.37	0.15	4.63	−0.56; 9.82	0.08	5.13 **	0.61; 9.61	**0.03**	3.07	−1.77; 7.92	0.21
	progress	−5.65	−12.84; 1.55	0.12	3.75	−3.27; 10.77	0.30	2.96	−3.16; 9.08	0.34	6.06	−0.49; 12.61	0.07
	unknown	−4.51	−11.99; 2.97	0.23	3.17	−4.13: 10.43	0.40	3.05	−3.32; 9.41	0.35	4.71	−2.09; 11.51	0.17
Treatment status	no vs. yes	−11.86 **	−17.12; 6.60	**<0.01**	4.34	−0.80; 9.48	0.10	7.46 **	2.98; 11.93	**0.001**	6.63 **	1.85; 11.41	**0.01**
Combined Treatments	surgery alone	reference											
	surgery + CT	−1.18	−7.48; 5.13	0.71	1.09	−5.06; 7.24	0.73	3.39	−1.94; 8.75	0.21	4.53	−1.20; 10.26	0.12
	surgery + RT	−2.79	−9.20; 3.61	0.39	5.74	−0.52; 12.00	0.07	4.12	−1.33; 9.57	0.14	4.32	−1.53; 10.17	0.15
	surgery + CT + RT	−2.64	−9.18; 3.90	0.43	4.36	−2.03; 10.75	0.18	7.93 **	2.36; 13.49	**0.01**	1.29	−4.66; 7.24	0.67
	CT alone	−0.71	−11.22; 9.80	0.89	4.28	−5.99; 14.56	0.41	10.28 **	1.34; 19.22	**0.02**	4.07	−5.56; 13.70	0.41
	all other	2.18	−6.43; 10.78	0.62	10.57 **	2.16; 18.98	**0.01**	5.50	−1.82; 12.82	0.14	−0.96	−8.78; 6.87	0.81
Time since diagnosis	0–<6 months	reference											
	6–<12 months	−2.20	−9.86; 5.45	0.57	5.27	−2.20; 12.74	0.17	3.68	−2.82; 10.18	0.27	1.31	−5.66; 8.27	0.71
	12–<24 months	1.30	−6.12; 8.73	0.73	0.66	−6.60; 7.91	0.86	4.16	−2.16; 10.48	0.20	7.51 **	0.73; 14.31	**0.03**
	24–<60 months	6.76	−0.26; 13.79	0.06	2.56	−4.31; 9.43	0.47	1.74	−4.25; 7.72	0.57	3.21	−3.20; 9.62	0.33
	60 months or more	4.84	−2.72; 12.40	0.21	0.13	−7.26; 7.53	0.97	0.80	−5.63; 7.24	0.81	4.80	−2.10; 11.70	0.17
Tumor recurrence	no	reference											
	yes	−6.41 **	−11.53; 1.30	**0.01**	6.31 **	1.32; 11.30	**0.01**	5.17 **	0.82; 9.52	**0.02**	0.87	−3.78; 5.52	0.71
	unknown	1.12	−11.40; 13.63	0.86	−1.60	−13.81; 10.6	0.80	−2.35	−12.99; 8.24	0.67	9.35	−2.21; 20.91	0.11

## Supplementary Materials

The following are available online at https://www.mdpi.com/2072-6694/12/12/3590/s1, Table S1: Sarcoma subtypes by histology, Table S2: Construction of sarcoma location variable, Table S3: Comparison study population with study participants without questionnaire and non-participants, Figure S1: Directed acyclic graphs of potential predictive variables of HRQoL of sarcoma patients. Yellow: recorded predictive variables, grey: variables not included in the model, blue: outcome. The absence of red arrows implies a model without uncontrolled confounding.
