# Supplementary material for: The Health-Related Quality of Life of Sarcoma Patients and Survivors in Germany—Cross-Sectional Results of a Nationwide Observational Study (PROSa)
Source: Cancers (Basel). 2020 Nov 30;12(12):3590. doi: 10.3390/cancers12123590 (PMC7759994; doi:10.3390/cancers12123590)
Supplement: Supplementary file 1 [file cancers-12-03590-s001.pdf]

Supplementary Materials

The Health-Related Quality of Life of Sarcoma Patients and Survivors in Germany—Cross-Sectional Results of A Nationwide Observational Study (PROSa)

Martin Eichler, Leopold Hentschel, Stephan Richter, Peter Hohenberger, Bernd Kasper, Dimosthenis Andreou, Daniel Pink, Jens Jakob, Susanne Singer, Robert Grützmann, Stephen Fung, Eva Wardelmann, Karin Arndt, Vitali Heidt, Christine Hofbauer, Marius Fried, Verena I. Gaidzik, Karl Verpoort, Marit Ahrens, Jürgen Weitz, Klaus-Dieter Schaser, Martin Bornhäuser, Jochen Schmitt, Markus K. Schuler and the PROSa study group

Includes Entities

We included sarcomas according to the following WHO classification.

- Fletcher CDM, World Health Organization, International Agency for Research on Cancer, editors. WHO classification of tumours of soft tissue and bone. 4th ed. Lyon: IARC Press; 2013. 468 p. (World Health Organization classification of tumours).
- Kurman RJ, International Agency for Research on Cancer, World Health Organization, editors. WHO classification of tumours of female reproductive organs. 4th ed. Lyon: International Agency for Research on Cancer; 2014. 307 p. (World Health Organization classification of tumours).
- Humphrey PA, Moch H, Cubilla AL, Ulbright TM, Reuter VE. The 2016 WHO Classification of Tumours of the Urinary System and Male Genital Organs—Part B: Prostate and Bladder Tumours. Eur Urol. 2016 Jul;70(1):106–19.
- World Health Organization, Swerdlow SH, International Agency for Research on Cancer, editors. WHO classification of tumours of haematopoietic and lymphoid tissues: [... reflects the views of a working group that convened for an Editorial and Consensus Conference at the International Agency for Research on Cancer (IARC), Lyon, October 25 - 27, 2007]. 4. ed. Lyon: Internat. Agency for Research on Cancer; 2008. 439 p. (World Health Organization classification of tumours).
- Moch H, Cubilla AL, Humphrey PA, Reuter VE, Ulbright TM. The 2016 WHO Classification of Tumours of the Urinary System and Male Genital Organs—Part A: Renal, Penile, and Testicular Tumours. Eur Urol. 2016 Jul;70(1):93–105.

Table S1. Sarcoma subtypes by histology.

Sarcoma groups	Subtypes (ICD-0)
Undifferentiated/ unclassified sarcoma	Sarcoma NOS (8800/3)
	Undifferentiated spindle cell sarcoma (8801/3) {1}
	Pleomorphic hyalinising angiectatic tumor (8802/1)
	Undifferentiated pleomorphic sarcoma (8802/3)
	Undifferentiated round cell sarcoma (8803/3)
	Undifferentiated epithelioid sarcoma (8804/3)
	Undifferentiated sarcoma, NOS (8805/3)
	Malignant fibrous histiocytoma (MFH)/ Malignant fibrous histiocytoma of the bone (8830/3)
Fibro- / myofibroblastic/ fibrohistiocytic tumors	Adult fibrosarcoma/ fibrosarcoma of the bone (8810/3)
	Myxoinflammatory fibroblastic sarcoma (8811/1)
	Myxofibrosarcoma (8811/3)
	Palmar/plantar Fibromatosis (8813/1)
	Infantile fibrosarcoma (8814/3)
	Solitary fibrous tumor (SFT)/ Hemangiopericytoma (8815/1)
	Solitary fibrous tumor, malignant (8815/3)
	Desmoid (8821/1)
	Abdominal fibromatosis (8822/1)
	Inflammatory myofibroblastic tumor (8825/1)
	Low grade myofibroblastic sarcoma (8825/3)
	Dermatofibrosarcoma protuberans (DFSP) (8832/1)
	Fibrosarcomatous DFSP (FS-DFSP) (8832/3)
	Pigmented DFSP (Bednar-Tumor) (8833/1)
	Giant cell fibroblastoma (8834/1)
	Low-grade fibromyxoid sarcoma (LGFMS, Evans-Tumor) (8840/3)
	Lipofibromatosis (8851/1)
	Plexiform fibrohistiocytic tumor (PFHT) (8835/1)
	Giant cell tumor of soft tissue (9251/1)
	Tenosynovial giant cell tumor, diffuse type (9252/1)
	Tenosynovial giant cell tumor, malignant (9252/3)
GIST	GIST, unknown malignancy (8936/1)
	GIST, malignant (8936/3)
Liposarcoma	Liposarcoma (NOS) / Liposarcoma of the bone (8850/3)
	atypical lipomatous tumor (8850/1)
	Myxoides liposarcoma (8852/3)
	Round cell liposarcoma (8853/3)
	Pleomorphic liposarcoma (8854/3)
	Mixed cell liposarcoma (8855/3)
	Dedifferentiated liposarcoma (8858/3)
	Well differentiated liposarcoma (8851/3)
Vascular tumors (other)	Angiosarcoma/ Angiosarcoma of the bone (9120/3)
	Composite hemangioendothelioma (9130/1)
	Epithelioid hemangioendothelioma (9133/3)
	Papillary intralymphatic angioendothelioma (9135/1)
	Retiform angioendothelioma (9136/1, 9137/1)
	Pseudomyogenic angioendothelioma (9138/1)
	Kaposi sarcoma (9140/3)
Leiomyosarcoma	Leiomyosarcoma/ Leiomyosarcoma of the bone/ Uterine leiomyosarcoma (8890/3)
	Epitheloid leiomyosarcoma (8891/3)
	Myxoid leiomyosarcoma (8896/3)
	Smooth muscle tumor of uncertain malignancy (8897/1)
Osteosarcoma	Osteosarcoma (NOS) (9180/3)
	Chondroplastic osteosarcoma (9181/3)
	Fibroblastic osteosarcoma (9182/3)
	Teleangiectatic osteosarcoma (9183/3)
	Small cell osteosarcoma (9185/3)
	Low grade osteosarcoma (9187/3)
	Parosteal osteosarcoma (9192/3)
	Periosteal osteosarcoma (9193/3)

	High Grade surface osteosarcoma (9194/3)<br>Secondary osteosarcoma
Synovialsarcoma	Synovial sarcoma, NOS (9040/3)<br>Synovial sarcoma, spindle cell (9041/3)<br>Synovialsarcoma, biphasic (9043/3)
Skeletal-muscle tumours (other)	Rhabdomyosarcoma/ pleomorphic Rhabdomyosarcoma NOS (8900/3)<br>Pleomorphic Rhabdomyosarcoma (8901/3)<br>Embryonal Rhabdomyosarcoma (8910/3)<br>Spindle cell/sclerosing Rhabdomyosarcoma (8912/3)<br>Alveolar Rhabdomyosarcoma (8920/3)
Nerve sheath tumours (other)	Ectomesenchymoma (8921/3)<br>Malignant peripheral nerve sheath tumor (MPNST) (9540/3)<br>Epithelioid MPNST (9542/3)<br>Melanotic schwannoma (9560/1)<br>Malignant Triton tumor (9561/3)<br>Malignant granular cell tumor (9580/3)
Ewing sarcoma	Ewing sarcoma (9260/3)<br>PNET (9473/3)
Chondrosarcoma	Chondroblastoma/ Chondrosarcoma Grade I (9220/1)<br>Chondrosarcoma Grade II/III (9220/3)<br>Juxtacortical chondrosarcoma (peripheral, periosteal) (9221/3)<br>Extraskeletal myxoides chondrosarcoma (9231/3)<br>Mesenchymal chondrosarcoma (9240/3)<br>Clear cell chondrosarcoma (9242/3)<br>Dedifferentiated chondrosarcoma (9243/3)
Stromasarcoma (other)	High-grade endometrial stromasarcoma (HG-ESS) 8930/3)<br>Low-grade endometrial stromasarcoma (LG-ESS) (8931/3)<br>Stromatumor, uncertain malignancy (8935/1)<br>Stromasarcoma (NOS) (8935/3)
Pericyclic (perivascular) tumors (other)	Malignant glomus tumor (8711/3)<br>Glomangiomatosis (8711/1)<br>Myofibromatosis (8824/1)
Tumours of uncertain differentiation (other)	Desmoplastic small round cell tumor (DSRCT) (8806/3)<br>Angiomatoid fibrous histiocytoma (8836/1)<br>Ossifying fibromyxoid tumor (OFMT), malignant (8842/3)<br>Angiomyolipoma (8860/1,2,3)<br>PEComa, NOS, malignant (8714/3)<br>Mixed tumor (NOS), malignant (8940/3)<br>Extra-renal rhabdoid tumor (8963/3)<br>Myoepithelial carcinoma (8982/3)<br>Phosphaturic mesenchymal tumor, malignant (8990/3)<br>Clear cell sarcoma of soft tissue (9044/3)<br>Sinunasal Glomangiopericytoma (9150/3)<br>Lymphangioleiomyomatosis (9174/1)<br>Peripheral primitive neuroectodermal tumor, PPNET (9364/3)<br>Parachordoma (9373/1)<br>Alveolar soft part sarcoma (9581/3)<br>Clear cell/sugar tumor of the lung<br>Intimal sarcoma (9137/3)
Bone sarcoma (other)	Hemangiopericytoma of the bone (8815/1)<br>Desmoplastic bone fibroma (8823/1)<br>Osteoklastoma NOS (9250/1)<br>Giant cell sarcoma (9250/3)<br>Adamantinoma (of the long tubular bones) (9261/3)<br>Chordoma (9370/3)<br>Chondroid chordoma (9371/3)<br>Dedifferentiated chordoma (9372/3)

Langerhans cell sarcoma (other)	Langerhans Cell sarcoma (9756/3)

Sarcoma Locations

Table S2. Construction of sarcoma location variable.

location	source question
abdomen/ retroperitoneum	Soft Tissue Sarcoma:<br>- abdomen unspecified<br>- retroperitoneum unspecified<br>- stomach<br>- small bowel<br>- large bowel<br>- rectum<br>- omentum<br>- peritoneum<br>- kidney<br>- liver<br>- spleen<br>GIST<br>- all types (stomach, small bowel, rectum, duodenum, esophagus, unspecified)
thorax	Soft Tissue Sarcoma:<br>- thorax unspecified<br>- lung<br>- pleura<br>- heart<br>- thoracic wall<br>Bone Sarcoma:<br>- thorax<br>- rib<br>- breastbone (sternum)
pelvis/ urogenital sarcoma	Soft Tissue Sarcoma:<br>- pelvis unspecified<br>- uterus<br>- ovary<br>- urinary bladder<br>- spermatic cord<br>- lesser pelvis<br>- gluteal region<br>Bone Sarcoma:<br>- pelvis unspecified<br>- haunch bone (os ilium)<br>- pubis<br>- seat bone (os ischium)<br>- acetabulum<br>- sacrum<br>- tailbone (os occygis)
lower limbs	Soft Tissue Sarcoma:<br>- upper leg<br>- lower leg<br>- groin<br>- feet<br>- ankle<br>- knee<br>Bone Sarcoma:<br>- thigh (femur)<br>- shinbone (tibia)<br>- calfbone (fibula)<br>- hind foot (tarsus)<br>- midfood (metatarsus)
upper limbs	Soft Tissue Sarcoma:<br>- shoulder girdle<br>- upper arm<br>- lower arm<br>- elbow

	- hand<br>Bone Sarcoma:<br>- upper arm bone (humerus)<br>- bladebone (scapula)<br>- collarbone (clavicula)<br>- ell or spoke (ulna or radius)<br>- metacarpal bones<br>- carpal bones
head&neck	Soft Tissue Sarcoma:<br>- head&neck unspecified<br>Bone Sarcoma:<br>- head&neck unspecified<br>- skull<br>- eye socket<br>- lower jaw<br>- upper jaw
back/ spine	Soft Tissue Sarcoma:<br>- back unspecified<br>- cervical region<br>- thoracic region<br>- lumbar region<br>- abdominal wall/ latus<br>Bone Sarcoma:<br>- spine
all other	Soft Tissue Sarcoma:<br>- extremities unspecified<br>- unknown site<br>Bone Sarcoma:<br>- extremities unspecified<br>- unknown site

Non-responder and non-participant analysis

To estimate possible selection bias, the analyzed study population was a) compared with the non-participants from whom corresponding data were available (N=231-278) and b) compared with the study participants from whom no questionnaires were available (N=196).

a) Non-participants were on average 4 years older than the analyzed population. Since increased age is associated with poorer quality of life, the values determined in the study may be slightly too positive. Regarding the variables gender and time since first diagnosis the values did not differ.

b) In the population of participants without a questionnaire, women were more frequently represented than men. This also indicates an error that could skew QoL values into the positive. The same applies to the smaller number of palliative patients. On the other hand, patients undergoing treatment are more strongly represented in the analyzed population, and the same applies to patients undergoing chemotherapy and radiotherapy. This may indicate that QoL values could be skewed to the negative.

Overall, the differences between the groups can be classified as secondary, especially since the majority of them indicate that the measured values are underestimated rather than overestimated.

Table S3. Comparison study population with study participants without questionnaire and non-participants.

Variable	Value	Study population %	Non-Respondents (no questionnaire) %	Non-participants %
Sex	female	48.7	57.3	49.3
Age at study inclusion (mean)		52.6	51.0	55.0
Time since diagnosis	0 - < 0.5 years	19.2	24.5	19.5
	≥0.5 - < 1 year	11.4	6.6	8.2
	≥1 - < 2 years	14.9	11.7	16
	≥2 - < 5 years	26.4	24.5	22.9
	≥5 years	28.2	32.7	33.3
Sarcoma Types	soft tissue sarcoma	70.5	65.3	
	bone sarcoma	17.8	19.9	
	GIST	11.7	14.8	
Tumor site	trunk	47.2	54.1	
	extremities	47.3	43.4	
	all other	5.5	2.6	
Aggressiveness of tumor	locally aggressive + rarely metastatic	7.8	10.7	
	malignant	92.2	89.3	
Treatment intention at study inclusion	palliative	24.0	26.6	
Disease status	complete remission	50.1	51.5	
	partial remission + stable disease	33.5	29.0	
	progress	16.4	19.5	
Treatment status*	in treatment	33.4	27.2	
Received treatment - surgery	yes	88.3	84.9	
Received treatment - chemotherapy	yes	47.9	40.8	
Received treatment - radiotherapy	yes	39.8	35.8	
Received treatment -TKI	yes	15.9	16.9	

Directed acyclic graphs

A model of directed acyclic graphs was used to develop the regression model and to identify possible predictive variables, possible confounders and intermediate variables (Figure S1).

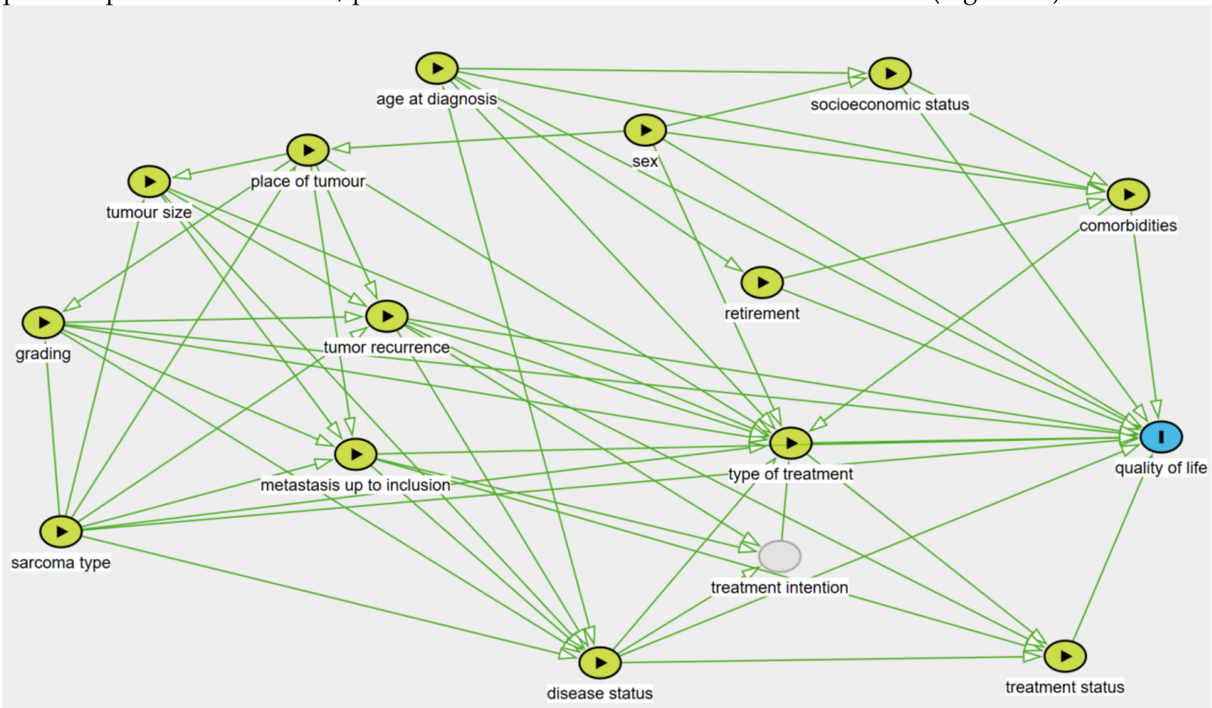

Figure S1. Directed acyclic graphs of potential predictive variables of HRQoL of sarcoma patients. Yellow: recorded predictive variables, grey: variables not included in the model, blue: outcome. The absence of red arrows implies a model without uncontrolled confounding.
